# Laboratory rearing of *Anopheles arabiensis*: impact on genetic variability and implications for Sterile Insect Technique (SIT) based mosquito control in northern Sudan

**DOI:** 10.1186/s12936-016-1484-2

**Published:** 2016-08-25

**Authors:** Rasha Siddig Azrag, Kamal Ibrahim, Colin Malcolm, Elamin El Rayah, Badria El-Sayed

**Affiliations:** 1 Tropical Medicine Research Institute, National Centre for Research, Khartoum, Sudan; 2 Department of Zoology, Faculty of Science, University of Khartoum, Khartoum, Sudan; 3 Department of Zoology, Southern Illinois University Carbondale, Carbondale, IL 62901 USA; 4 School of Life and Medical Sciences, University of Hertfordshire, Hatfield, UK

**Keywords:** *Anopheles arabiensis*, Mosquitoes, Colonization, Genetic variability, Microsatellites, Sterile insect technique

## Abstract

**Background:**

Mosquito colony populations often show significant changes in their population genetic make-up compared to the field populations that were used as founding source. Most of the changes that have been reported are indicators of depletion in the overall genetic diversity of the colony populations. The Sterile Insect Techniques programme of mosquito control that is underway in Northern Sudan uses sterilized males produced from a laboratory-maintained colony population. The genetic diversity of an advanced generation of this colony population was quantitatively assessed and compared to the field population from which the colony was derived.

**Methods:**

*Anopheles arabiensis* mosquito samples from the 13th generation of the colony, and from the locality that was the source of the first generation of the colony, were genotyped at 11 microsatellite loci distributed throughout the species’ genome. Standard population genetic analyses were carried out to quantify and compare their population genetic make-up and diversities.

**Results:**

The colony samples showed significant reduction in the total number of alleles, the numbers of rare and private alleles, and the fractions of heterozygote individuals at all the loci. The pattern of change is consistent with the expected effect of the use of a small number of mosquitoes when the colony was established. Departure from Hardy–Weinberg equilibrium in the direction of homozygote excess was observed at some loci and attributed to the presence of null-alleles.

**Conclusions:**

This study highlights the need for broad sampling when initiating colony populations and for ongoing assessment of the population genetic make-up of colony populations. Previous assessments of survivorship, dispersive behaviour and swarm formation indicate that the inbreeding and reduced genetic variability reported in this study may not have had direct fitness consequences yet. However, noting the lessons learned in other SIT programmes about the impact of colonization on male sexual behaviour and longevity, as well as other inbreeding related adverse effects, a systematic investigation of these potential effects is recommended because they have direct impact on the ultimate success of the programme.

## Background

Insect species are reared in laboratories to produce colony populations from which a steady supply of the desired life-stage can be obtained for research as well as for biological control purposes. The laboratory colonies are either representative of the field populations from which they originate or serve as a stable standard to which other populations may be compared. Many studies have examined the genetic consequences of artificial rearing when compared to wild populations; some with focus on the implications on using the laboratory animals in pathogen transmission studies. These studies have revealed that the genetic make-up of colony populations often shows significant departures from the originally sampled field populations. Common features of the departures include: reduced number of alleles, divergence in allele frequency, presence of new alleles, reduced heterozygosity, fewer multi-locus genotypes, fewer rare alleles, and, reduced number of private alleles. Most of the changes that have been reported are indicators of depletion in the overall genetic diversity [1–4]. The consequences of inbreeding on reproductive traits of laboratory strains overtime has also been examined [5].

Some of the common genetic changes that accompany laboratory rearing of insect colonies are not universal. A substantially lower rate of fixation at several enzyme loci in inbred stocks of the tree-hole mosquito, *Aedes triseriatus,* was reported and it was concluded that lethality of homozygous recessives at these loci accounts for the elevated heterozygosity [6]. Similarly, [7] were able to demonstrate that inbred lines of the *Anopheles gambiae* sensu stricto retained discrete genomic blocks that maintained high heterozygosity due to polymorphic chromosomal inversions. Broadly speaking, comparisons of the genetics of colony populations and field populations is important particularly in genetic control trials because population genetic make-up can affect physiological and behavioural fitness traits, and hence the efficacy of the genetic control methods [8–10]. The fitness of *Anopheles arabiensis* male populations in SIT trials in northern and south Africa, showed that prolonged colonization, irradiation, and transportation do not impede mating vigour and competitiveness of male mosquitoes [11–14]. Colonization of *An. arabiensis* under semi-field conditions was also shown to be associated with the retention of a higher degree of genetic diversity, reduced inbreeding and greater phenotypic similarity to the founding wild population than observed in laboratory-based small cage colonies [15].

This study assessed the allelic and genotypic diversity of the *An. arabiensis* Patten, 1905, colonies that are maintained at the Tropical Medicine Research Institute (TMRI) in Khartoum, Sudan. The aim was to explore the implications of any changes in the genetic makeup of the colony population to the ongoing trials of Sterile Insect Technique (SIT) based mosquito control in northern Sudan.

## Methods

### Source population

The mosquito species *An. arabiensis* is the only member of the *Anopheles gambiae* species complex that is found in northern Sudan and it is the major malaria vector in the region. Mosquito larvae were collected from the Kabtoad area of Dongola town, from water pools along the banks of River Nile [12]. Larvae were collected from different habitats including leaking water pipes, grassy river banks and stagnant water in potholes using the classical dipping method [16]. The larvae were cleaned from predators and other *Culex* mosquito larvae, pooled together and transported in plastic containers to the insectary at the TMRI in Khartoum where they were reared. Sample sizes from each habitat type varied; when pooled, there were sufficient larvae to stock 3 white dishes with a minimum of 300 larvae each as described below.

### Laboratory rearing

The TMRI insectary, established in 2002, maintains a controlled environment of between 17 and 20 °C temperature and relative humidity of 80 %. The mosquito larvae were placed in large white dishes (30 cm × 20 cm), kept under light for 10 h per day and fed commercial baby food. The larvae were kept in the water from their larval habitat; most pupated without adding food. Pupae were collected using a dropper and transferred into 30 cm × 30 cm cages. Emergent adults were fed on glucose sugar (5 %). Subsequently, adult mosquitoes were fed human or rabbit blood at sunset and at night. Successive generations were reared in this manner. The main food for larvae for subsequent generations was baby food (Nestle, Cerelac). The pupae of the first generation of eggs reared in the laboratory were transferred into cages labelled G1 and reared under the same laboratory conditions. Single adult flies were collected from these cages using an aspirator, replaced in −20 °C for 2 min to knock down the mosquito then quickly single mosquitoes were placed in a labelled cryotube and preserved in 90 % ethanol at −20 °C for microsatellites analysis. All larval dishes were covered with fine mesh. The average duration of each colony generation was around 3 weeks. However, the early generations, especially G1–G3 required longer time due delayed egg hatching or restocking of adult samples. This extended the duration of the colony establishment phase.

### DNA extraction

DNA extraction from individual insects was carried out using a potassium acetate lysis buffer followed by alcohol precipitation as described in [17]. The extracted DNA was dissolved in 100 μl of T.E., pH 8.0. and stored at −20 °C until used in PCR amplification.

### Species confirmation

PCR using species-specific primer pairs was used to confirm identification. The universal mosquito primer UN-5′GTGTGCCCCTTCCTCGATGT3′ was used as a forward primer, paired with the *Anopheles gambiae* specific reverse primer GA-5′CTGGTTTGGTCGGCACGTTT3′ or the *An. arabiensis* specific reverse primer: AR-5′AAGTGTCCTTCTCCATCCTA3′ according to [18]. The PCR cocktail contained 1.2 µM of each of the four dNTPs, 0.5 units Tag polymerase, 0.5 µM each forward and reverse primers made up to a total volume of 25 µl with PCR buffer containing 1 mM MgCl. The PCR was carried out in an ABI 97000 Gene Amp thermal cycler as follows. Initial denaturing for 4 min at 94 °C followed by 30 cycles of denaturation at 94 °C for 30 s, annealing at 50 °C for 30 s and extension at 72 °C for 30 s, followed by a final extension for 5 min at 72 °C.

### Microsatellite analysis

Eleven primer pairs that amplify microsatellite loci located on autosomal chromosomes 2 and 3, and the X chromosome as described in [19] and [20] were used. The primer sequences, their annealing temperatures, previously reported expected modal allele size and the repeat motif are shown in Table 1.

**Table 1:** The microsatellites loci used in the study [9, 10]

Locus	Foreword primer	RM	Reverse primer	A_S_
AgXH7	CACGATGGTTTTCGGTGTGG	(GT)8	ATTTGAGCTCTCCCGGGTG	99
AgXH180	GTATGTTGTGATCTCCTGCC	(GT)10	AAAACGAGCCACCACCAGAG	72
Ag2H175	AGGAGCTGCATAATTCACGC	(CA)8	AGAAGCATTGCCCGCATTCC	97
Ag2H1010	GCGTATGTCAATGGCGAGAA	(GATA)6	CGCTGGAAATTGTCACACC	117
Ag2H46	CGCCCATAGACAACGAAAGG	(GT)8	TGTACAGCTGCAGAACGAGC	138
Ag2H26	GGTTCCTGTTACTTCCTGCC	(GT)8	CCGGCAACACAAACAATCGG	154
Ag2H143	CGTACGAGTGAGTGAGTTGG	(TC)9	CAAAAATAGCATCACGGCCG	160
Ag3H249	ATGTTCCGCACTTCCGACAC	(GT)15	GCGAGCTACAACAATGGAGC	129
Ag3H88	TGCGGCGGTAAAGCATCAAC	(GT)9	CCGGTAACACTGCGCCGAC	176
Ag3H93	5′ 8TCCCCAGCTCACCCTTCAAG3′	(GT)4 + 7	3′GGTTGCATGTTTGGATAGCG5′	209
33C1	5′8TTGCGCAACAAAAGCCCACG3′	(AGC)6	3′ATGAAACACCACGCTCTCGG5′	159

*RM* repeat motif, *A*_*T*_ annealing temperature, *A*_*S*_ expected allele size in bp

Microsatellite genotyping was carried out according to [21]. The forward primer of each locus was 5′ labelled with a FAM, HEX or NED fluorescent tag, followed by PCR amplification as shown in the species confirmation protocol above, except for an annealing temperature of 55 °C. PCR products were scanned in an ABI PRISM 3700 sequencer (Applied Biosystem) following manufacturer’s protocols. The Genotyper DNA Fragment Analysis Software (Applied Biosystems) was used to call allele sizes.

### Data analysis

The software package Tandem [22] was used for binning allele sizes. The output file from Tandem was used as input file in Convert [23] to obtain data files for use in the population genetic software packages Web Genepop [24] and Arlequin [25]. The latter were used to obtain allele and genotype frequencies and to test for deviation from Hardy–Weinberg equilibrium (HWE). The AmCharts JavaScript library was used to draw nested pie charts depicting the frequency distributions of the alleles at the nine linkage group 2 and 3 loci.

## Results

### Allele sizes

A total of 58 *An. arabiensis* mosquitoes collected from Dongola area, henceforth referred to as field population, were compared to 51 samples from generation 13 of the *An. arabiensis* colony maintained at the TMRI insectary, referred to as colony population. DNA from each sample was PCR amplified using the species specific primers to confirm species identification and subsequently genotyped at 11 microsatellite loci, nine of which have been mapped to linkage groups (chromosomes) 2 and 3 while two are on the X chromosome [20]. Binned allele sizes at all the loci varied by multiples of the respective repeat motifs shown in Table 1.

### Allele frequencies and rare alleles

**Fig. 1:**
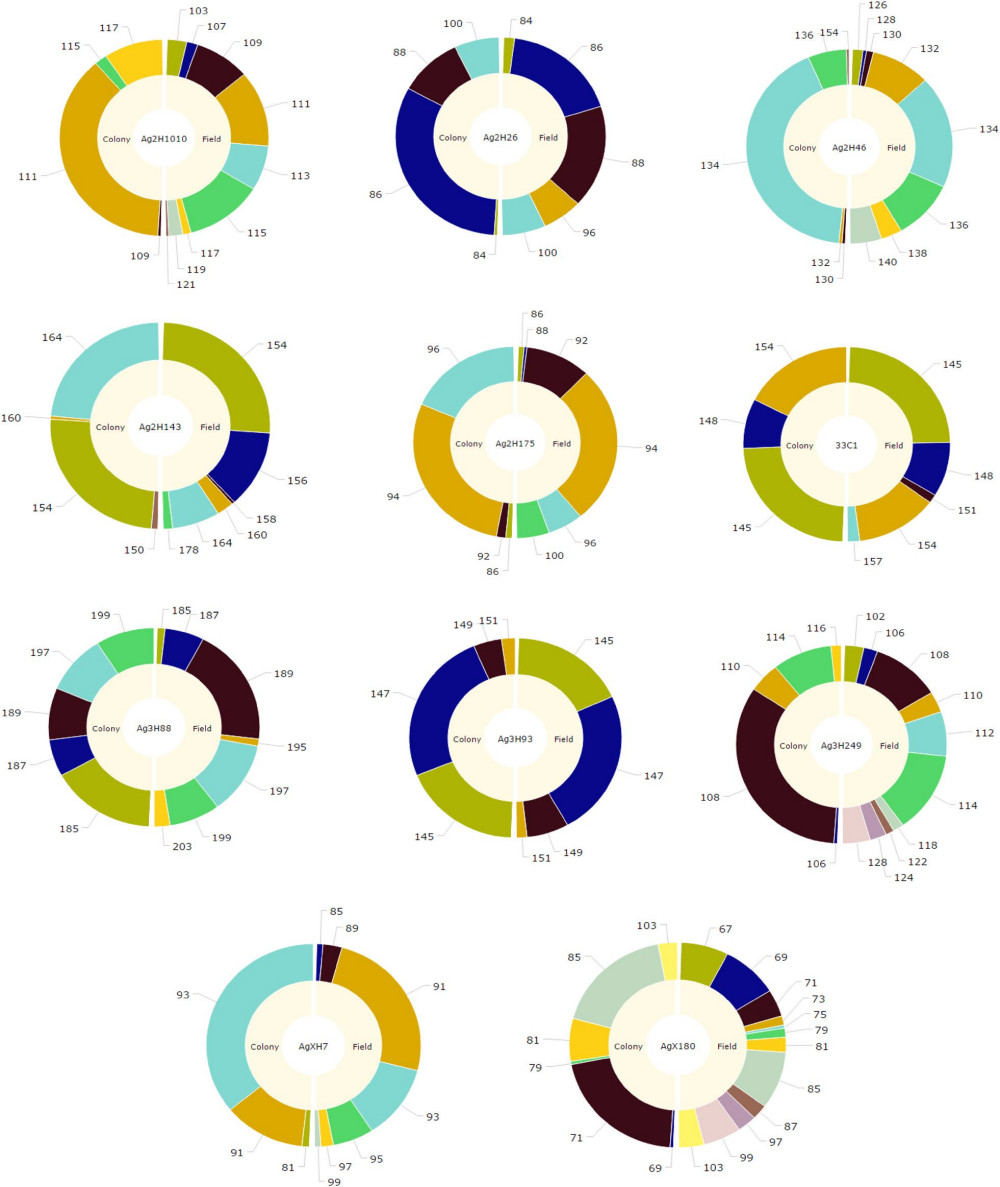
Frequency distribution of microsatellite alleles in the colony (*left half* of pie chart) and field (*right half*) populations of *Anopheles arabiensis*. Allele sizes in base pairs are shown as labels of the pie-chart segments; segments of the same color correspond to the same allele in the colony and field populations

Figure 1 compares allele frequencies at the 11 microsatellite loci in the field and colony populations. The donut plots for all but one of the 11 loci clearly show a dramatic reduction in the number of alleles in the colony population (left half) compared to the source population from Dongola (right half). Only one locus, Ag3H93, retained the same number of alleles in both populations (Table 2). We note that in all the loci where allele numbers declined, the commonest allele retained in the 13th generation of colony population is either the commonest or one of the common alleles in the field population. The average number of alleles per locus in the colony population (4.27) was reduced by 40 % compared to the field population (7.27). These are the expected trends under a scenario of divergence from the source that is due to founder effect coupled with subsequent drift during the 13 generations of colony maintenance. All tests of genic differentiation, i.e. test of the statistical significance of the allele frequency divergence between the two populations, were highly significant (p < 0.001). The number of rare alleles, defined as alleles with frequencies of less than 5 % in either population, is another contrasting feature between the wild population and the laboratory colony. There were a total of 29 rare alleles in the former, an average of 2.64 per locus. The corresponding estimates for the colony population were 16 alleles, an average of 1.45 alleles per locus.

**Table 2:** Genetic variability comparisons between the field and colony populations

Locus	Tot. N_a_	Field						Colony					
		N (N_a_)	A_E_ (P_A_)	Heterozygotes		F_IS_	P value (HWE)	N (N_a_)	A_E_ (P_A_)	Heterozygotes		F_IS_	P value (HWE)
				Obs.	Exp.					Obs.	Exp.		
Ag2H1010	9	57 (9)	5.60 (5)	27	46.82	0.4255	*0.0000*	51 (4)	1.62 (0)	22	19.51	−0.1288	0.7020
Ag2H175	6	57 (6)	2.78 (2)	32	36.53	0.1252	0.0260	50 (4)	2.13 (0)	30	26.53	−0.1321	0.6718
Ag2H26	5	44 (5)	3.63 (1)	36	31.87	−0.1312	0.6019	50 (4)	2.10 (0)	30	26.15	−0.1489	0.7849
Ag2H46	9	45 (8)	4.42 (4)	17	34.83	0.5148	*0.0000*	49 (5)	1.37 (1)	14	13.23	−0.0583	0.3975
Ag2H143	7	53 (6)	2.88 (3)	37	34.59	−0.0704	0.2976	51 (4)	2.14 (1)	24	27.19	0.1186	0.0349
Ag3H249	11	58 (10)	6.48 (6)	41	49.05	0.1654	0.0201	46 (5)	2.03 (1)	19	23.29	0.1861	0.0194
Ag3H88	7	41 (7)	4.16 (2)	14	31.14	0.5536	*0.0000*	41 (5)	4.66 (0)	32	32.20	0.0066	0.0880
33C1	6	52 (6)	3.05 (2)	36	34.47	−0.0450	0.2890	51 (3)	2.65 (0)	40	31.77	−0.2622	0.0274
Ag3H93	4	46 (4)	2.71 (0)	27	29.04	0.0870	0.8145	46 (4)	2.57 (0)	28	28.08	0.0032	0.9470
AgXH7	8	52 (7)	3.10 (5)	19	35.23	0.4632	*0.0000*	44 (3)	1.70 (1)	6	18.16	0.6720	*0.0000*
AgX180	12	52 (12)	8.92 (6)	21	46.17	0.5476	*0.0000*	51 (6)	3.08 (0)	18	34.42	0.4795	*0.0000*
Mean	7.64	50.64 (7.27)	4.34 (3.27)	–	–	–	–	(4.27)	2.37 (0.36)	–	–	–	–

P < 0.01 considered as statistically significant and shown in italics

*Tot* total, *N*_*a*_ number of alleles, *N* sample size, the number of mosquitoes successfully genotyped at each locus, *A*_*E*_ effective number of alleles, *P*_*A*_ number of private alleles, *Obs* observed number of heterozygotes, *Exp* expected number of heterozygotes, *F*_*IS*_ inbreeding coefficient, *HWE* Hardy–Weinberg Equilibrium

### Private alleles and effective number of alleles

A private allele is an allele present in one, but not the other of the two populations that were compared. The total number of private alleles combined across all loci was 40, of which 36 were private to the field population. Averaged across all 11 loci, the number of private alleles in the field population was almost ten times as many as that in the colony population, 3.27 vs 0.36 private alleles per locus (Table 2). This is further evidence of the significant reduction in genetic diversity experienced by the colony population at founding and during the 13 generations of insectary propagation.

The effective number of alleles (A_E_) is a measure of allele diversity, which is used when comparing populations in which the number of alleles and their frequency distributions differ drastically. It is defined as the number of equally frequent alleles it would take to achieve a given gene diversity, represented by the expected heterozygosity (H_e_) of a population. It is computed as A_E_ = (1 − (1/H_e_). Again, a consistent trend of reduction in the effective number of alleles at all but one locus is evident in the colony population compared to the field population (Table 2). Average A_E_ in the colony population has been reduced by 45 %. This is comparable to the reduction in the raw allele count; it appears that the difference in the number of alleles per locus contributes more to the divergence between the two populations than does the difference in allele frequencies.

### Genotype frequencies, Hardy–Weinberg equilibrium

The average number of heterozygotes per locus is higher in the field population reflecting the higher number of alleles per locus. Furthermore, in both the field and the colony populations, where statistically significant, the deviations from HWE expectations were always in the direction of homozygote excess (Table 2). The colony samples were at HWE at all the autosomal loci; the field population showed significant homozygote excess in three of them as did both populations at the two X-linked loci.

## Discussion

This study confirms that microsatellite allelic and genotypic diversities have declined considerably in the TMRI colony of mosquitoes compared to the field population from which it was established. Comparable loss of diversity has been reported in Mali by Norris et al. [3] at nine microsatellite loci in two colonies of *Anopheles gambiae* compared to a field population. Baeshen et al. [5] also reported significant reduction in genetic diversity and increase in homozygosity in a similar study involving *Aedes triseriatus* populations. The mostly likely causes of the observed changes are discussed below, followed by their implications to the sterile insect techniques (SIT) based mosquito control programme underway in Northern Sudan that uses the TMRI population as source of sterile male mosquitoes.

The contrasting features of the genetic diversities of the wild and colony populations, taken individually or combined, could be due to the founder effect. When a new population is founded from a small, random sub-sample of a genetically diverse population the common alleles in the source population are much more likely to be sampled than the rarer alleles, and, there is bound to be a reduction in diversity. This is borne by the data in that the common alleles retained in the colony population are also common in the field population (Fig. 1). The reduction in the total number of alleles, the private alleles and the rare alleles is also most likely to be the outcome of founder effect. Drift during the 13 generations of propagation in the laboratory could have contributed, but the size of the colony population in successive generations, which was significantly bigger than the typical population size at a breeding site, was large enough to minimize this. Thus, it is concluded that limited sampling during colony establishment contributed most to the contrasting patterns of genic diversity of the field and colony populations. The fact that Hardy–Weinberg genotypic proportions were confirmed at all the autosomal loci in the colony population implies that the reduction in average heterozygosity compared to the field population is due to the reduction in the number of alleles. On the other hand, the homozygote excess at three autosomal loci in the field population and the two X-linked loci in both populations is attributable to the presence of null-alleles. Other potential evolutionary causes of excess homozygosity at loci that are presumed neutral are expected to affect most if not all 11 loci.

A number of recent studies comparing colonization of *An. arabiensis* under semi-field conditions to laboratory-rearing in small cages have reported that the former retained a higher level of genetic diversity, showed less inbreeding and were phenotypically more similar to the founding wild population [5, 15, 26]. The cage-reared TMRI colony population falls in the latter category. Reduction in genic diversity and increase in homozygosity could depress fitness as a result of the expression of deleterious recessive alleles combined with the loss of heterozygote advantage. This trend has been confirmed using pedigree-based breeding experiments, which showed a decline in fitness-associated traits in individuals with high inbreeding coefficients [27–29]. However, the generality of the heterozygosity-fitness correlations in natural populations is difficult to assess, because most studies that have evaluated the relationship in the wild are either experimental or performed on smaller isolated populations [30–32].

All novel insect control strategies involving mass release must address the issue of the evolutionary trajectory of genetic diversity in the released control agents relative to the source. This study has not confirmed or excluded the possibility that the elevated inbreeding in the colony population compared to the source population has led to loss of mating vigour in the sterile males, or any other change in their reproductive characteristics, or the evolution of premating isolation mechanisms. However, relevant insight has been gained from other studies focusing on the impact and consequences of colonization, mass rearing and loss of diversity within the SIT trial programme in northern Sudan. Excoffier and Lischer [25], who studied the capacity of released sterile *An. arabiensis* males to survive, disperse and participate in swarms occurring at varying distances from the release site, showed that sterile *An. arabiensis* males released into the field were able to find and participate in existing swarms. Helinski et al. [11] and Hassan et al. [12] reported no major obstacles associated with the small-scale irradiation and transportation of *An. arabiensis* males in the current SIT setting and laboratory-reared and irradiated *An. arabiensis* males from sixty generations were able to inseminate wild females at rates comparable to wild males. Mating competitiveness experiments showed that irradiated male mosquitoes are fertile as wild counterparts under semi-field conditions. However, they were not as competitive under laboratory conditions [14].

## Conclusions

This study highlights the need for broad sampling when initiating mosquito colony populations. Ongoing assessment of the population genetic make-up of colony populations at regular intervals is also recommended. The SIT programme in Northern Sudan has an active research programme that has looked into survivorship, dispersive behaviour and swarm formation in the colony population we studied. These studies have indicated that the inbreeding and reduced genetic variability we report may not have had direct impact on the traits that were investigated. However, we note the lessons learned in other SIT programmes about the impact of the population genetic makeup of release populations on male sexual behaviour [33], on the relative longevity of sterile males [34], and, on the extent to which inbreeding related adverse effects may be mitigated by occasional outbreeding of colony populations with wild caught mosquitoes [5]. A systematic investigation of these potential consequences of loss of genetic diversity is recommended because they have direct and significant impact on the ultimate success of the SIT programme in the Sudan.

## Abbreviations

IAEA: International Atomic Energy Agency
SIT: Sterile Insect Technique
TMRI: Tropical Medicine Research Institute—Khartoum, Sudan
